# The Mechanisms of Chansu in Inducing Efficient Apoptosis in Colon Cancer Cells

**DOI:** 10.1155/2013/849054

**Published:** 2013-05-30

**Authors:** Chun Li, Saeed M. Hashimi, Siyu Cao, Albert S. Mellick, Wei Duan, David Good, Ming Q. Wei

**Affiliations:** ^1^Division of Molecular and Gene Therapies, Griffith Health Institute and School of Medical Science, Griffith University, Gold Coast, QLD 4222, Australia; ^2^School of Medicine, Deakin University, Waurn Ponds, VIC 3220, Australia; ^3^School of Physiotherapy, Australian Catholic University, Banyo, QLD 4014, Australia

## Abstract

Chansu is one of the most widely used traditional Chinese medicines in China, Japan, and other Southeast Asian countries primarily for antipain, anti-inflammation, and recently anticancer. Over 10 recipes and remedies contained Chansu, which are easily available in pharmacies and hospitals, but the mechanisms of action were not clearly articulated. In the present study, Cinobufagin (CBF), the major compound of Chansu, was employed as a surrogate marker to determine its ability in inducing cancer cell death. As expected, CBF has significant cancer-killing capacity for a range of cancers, but such ability differs markedly. Colon and prostate cancers are more sensitive than skin and lung cancers. Interestingly, cancer cells die through apoptotic pathway either being biphasic caspase-3-dependent (HCT116) or independent (HT29). Multipathway analysis reveals that CBF-induced apoptosis is likely modulated by the hypoxia-inducing factor-1 alpha subunit (HIF-1**α**) as its inhibition was evident *in vitro* and *in vivo*. Taken together, these results demonstrate that CBF is a potent apoptotic inducer with potential for further development as a novel and effective anticancer agent for a range of cancers, especially colon cancer.

## 1. Introduction

In the last decade, considerable efforts have focused on improving cancer chemotherapeutics because conventional therapies fail to manage cancer effectively without causing significant side effects on patients [1–5]. There is a notion that a “chemotherapy efficacy plateau” has been reached [5, 6]. Since traditional herbal medicines have been used successfully for hundreds of years, potential novel anticancer compounds are being eagerly sought from them. Extensive research on examining the ability of natural compounds in inducing apoptosis, and with it the mechanisms of action has been reported [7–10].

Traditional Chinese medicine Chansu is a dried product of glandular secretion from the Asiatic toad (*Bufo gargarizan*). It has been used for centuries in various prescriptions, recipes, and ready-available remedies, such as Liushenwan (anti-inflammation), Shexiangbaoxinwan (antiarrhythmia), and Niuhuangxiaoyanwan (anti-inflammation and anaesthesia) in China, Japan, and other Southeast Asian countries [11, 12]. Recently, Chansu has been used as significant anticancer agents, amending the life quality of cancer patients [13].

Major components in Chansu include a group of bufadienolides, including Cinobufagin (CBF), Bufalin, and Resibufogenin [14]. CBF accounts up to six to seven percent of Chansu. Limited research on Chansu and its components has shown proapoptotic functions [14–17] and an inhibitive effect on a wide range of cancer cells [18–21]. Kamano et al. [22] reported that CBF has a potent inhibitory capacity against primary liver carcinoma cells. CBF has also been found to belong to the family of cardiac glycosides (CGs) and inhibit Na^+^/K^+^-ATPases activity [23], which emerged recently as novel drugs in prevention of cancer proliferation without serious side effects [24].

In this study we aimed to explore the capacity of CBF in inhibiting cancer cell proliferation and the mechanisms of such an effect. The understanding of the intracellular activities of CBF would assist us to develop the natural compound into a novel agent for cancer therapy.

## 2. Experimental

### 2.1. Human Cell Lines and Culture Conditions

Human cancer cell line HCT116 (colon), HT29 (colon), A431 (skin), PC3 (prostate), A549 (lung), and MCF-7 (breast) were purchased from American Tissue Culture Collection. Human lung cancer cell line Spc-A_1_ was purchased from Cell Bank of Chinese Academy of Sciences. All the cancer cell lines were cultured in Dulbecco's modified essential medium (Gibco, life technologies, VIC, Australia) supplemented with 10% foetal bovine serum (HyClone, Thermo Fisher Scientific, VIC, Australia), 100 U/mL penicillin (Sigma, Sigma-Aldrich, NSW, Australia), 100 *μ*g/mL streptomycin (Sigma), 110 mg/L sodium pyruvate (Gibco), and 25 mM HEPES (Gibco), in an atmosphere of 5% CO_2_ and 95% air at 37°C. To culture cells under hypoxic conditions, flasks containing cell lines were placed in a GasPak EZ pouch (BD, MD, USA) or were exposed to 100 *µ*M CoCl_2_ (Sigma). 

### 2.2. Cytotoxicity Assays

Cytotoxicity of CBF in all cell lines was studied by using 3-(4,5-dimethylthiazol-2-yl)-2,5-diphenyltetrazolium bromide (MTT) assays (Molecular Probes, life technologies, OR, USA). After the 24 hours starvation in serum-free culture, cells (1 × 10^4^ cells per a 96 well) were exposed to CBF and/or *N*-Acetyl-L-cysteine (NAC) treatment for another 24 or 48 hours, and then MTT reagent was added following the manufacturer's instructions. The absorbance was read at 570 nm and EC_50_ values were calculated by Prism 5 (GraphPad Software, CA, USA). CBF and NAC were purchased from Sigma and dissolved in dimethyl sulfoxide.

### 2.3. Detection of Annexin V/Propidium Iodide (PI) Apoptosis

HCT116 and HT29 cells were treated with 1 *μ*M CBF or 0 *μ*M of CBF as a negative control for 24 hours. The cells were harvested and stained with an AlexaFluor 488 Annexin V/Dead cell apoptosis kit (Molecular Probes). Experiments were performed according to the manufacturer's instructions. 

### 2.4. Analysis of Mitochondrial Potential Changes

HCT116 and HT29 cells (1 × 10^4^ cells per a black *μ*Clear 96 well (Greiner Bio-One, Frickenhausen, Germany)) were treated with 1 or 0 *μ*M of CBF. 5,5′,6,6′-tetrachloro-1,1′,3,3′-tetraethylbenzimidazolocar-bocyanine iodide (JC-1) dye (Sigma-Aldrich, MO, USA) was added to the cells at 3, 6, 9, 12, 15, 18, 24, and 48 hours. The experiment was carried out following the protocols provided by the manufacturer. At different time points, both red fluorescence and green fluorescence were recorded using POLARstar Omega (BMG Labtech, VIC, Australia).

### 2.5. Multipathway Reporter Assays

This multipathway activity assay was performed using Cignal Finder Toxicity 10-Pathway Reporter Arrays (SABiosciences, Qiagen, VIC, Australia) according to the manufacturer's instructions. Reporter and control DNA constructs of 10 selected transcription factors were transfected into HCT116 cells (3 × 10^4^ cells per a 96 well) using Lipofectamine LTX reagent (Invitrogen, life technologies, VIC, Australia). Twenty-four hours later, cells were treated with 1 *μ*M CBF for 9 hours. The luciferase assays were then carried out by using Dual-Glo luciferase assay systems (Promega, WI, USA).

### 2.6. Immunofluorescent Staining

For *in vitro *experiments, cells were grown on sterile 15 mm coverslips (1.5 × 10^5^ cells/coverslip/well) in a 24-well plate. After CBF treatment, cells were blocked in block buffer (3% normal goat serum and 0.5% BSA in 0.01 M PBS) for 30 min. For *in vivo*, fixed tissues were blocked in the same block buffer. Cells or tissues were sequentially incubated with corresponding primary antibodies against active caspase-3 (1 : 100; Abcam, Sapphire Bioscience, NSW, Australia), AIF (1 : 100; Cell Signalling, MA, USA), or HIF-1*α* (1 : 100; Novus, CO, USA), followed by incubation with a secondary antibody FITC or Texas Red-conjugated goat anti-rabbit IgG (1 : 2000; Abcam). The coverslips or tissues were then counterstained with DAPI (Molecular Probes) and treated with ProLong Gold antifade reagent (Molecular Probes). After 24 hours, fluorescence images were visualised using confocal microscopy (FV1000, Olympus, QLD, Australia).

### 2.7. Caspase-3/7 Activity Assays

Cells (1 × 10^4^ cells per a white *μ*Clear 96 well (Greiner Bio-One)) were exposed to CBF for 24 hours. The activity of caspase-3 was evaluated using Caspase-Glo 3/7 assay systems (Promega) according to the manufacturer's instructions. Luciferase intensity was measured by POLARstar Omega at 4, 8, 12, and 24 hours after CBF addition.

### 2.8. Real-Time RT-PCR

Total RNA was isolated from HCT116 and HT29 cells after 12 and 24 hours CBF treatment using Purelink RNA Mini Kit (Ambion, life technologies, CA, USA). RNAs in mouse tissues were initially stabilised by immersing in RNA*later* (Ambion) and extracted using Trizol reagent (Ambion). To synthesize first-strand cDNA, SuperScript III Reverse Transcriptase (Invitrogen) was used according to the manufacturer's instructions. Real-time PCR was carried out in a 20 *μ*L of reaction solution, with Express SYBR GreenER qPCR SuperMix (Invitrogen). No genomic DNA contamination or pseudogenes were detected. Primers used in real-time PCR were Human AIF (Forward: 5′-CTG AAA GAC GGC AGG AAG GTA G-3′, Reverse: 5′-CTC CAG CCA ATC TTC CAC TCA C-3′). Human caspase-3 (Forward: 5′-GTT TGT GTG CTT CTG AGC CAT G-3′, Reverse: 5′-CCA CTG TCT GTC TCA ATG CCA C-3′). Human HIF-1*α* (Forward: 5′-AAG GTA TTG CAC TGC ACA GGC-3′, Reverse: 5′-CAG CAC CAA GCA GGT CAT AGG-3′). Human GAPDH (Forward: 5′-GTC TCC TCT GAC TTC AAC AGC G-3′, Reverse: 5′-ACC ACC CTG TTG CTG TAG CCA A-3′).

### 2.9. Western Blotting

CBF-treated cells were scraped and lysed in cold lysis buffer, consisting of 25 mM of Tris (pH7.4), 125 mM of NaCl, 2.5 mM of EDTA, 25 mM of NaF, 0.5 mM Na_3_VO_4_, 0.5% Nonidet-P40, and 0.01% NaN_3_, supplemented with 1mM PMSF (Sigma) and protease inhibitor cocktail (Sigma). Mouse tissues (<125 mm^3^) were initially ground by mortar and pestle, followed by the immersion in lysis buffer. After SDS-PAGE and PVDF transfer, protein samples were incubated with primary antibodies against AIF (1 : 1000), active caspase-3 (1 : 1000), HIF-1*α* (1 : 500), or *α*-Tubulin loading control (1 : 2000; Abcam) overnight at 4°C, followed by incubating with horseradish peroxidise-conjugated goat anti-mouse or anti-rabbit (1 : 7500; Abcam) antibodies. SuperSignal chemiluminescent substrate (Pierce, IL, USA) was finally added, and the chemiluminescence was visualised using a VersaDoc 4000MP system (Bio-Rad, CA, USA).

### 2.10. Transient Transfection Assays

Vectors encoding full-length human HIF-1*α* and pEGFP-C1 (Clontech, CA, USA) were kindly provided by Dr. Zhou Wang and Associate Prof. Steve Ralph, respectively. The sequence of full-length HIF-1*α* was amplified by PCR and inserted in a pEGFP-C1 vector, which generated pGFP-HIF-1*α*. Plasmid pGFP-HIF-1*α* was transfected into HCT116 and HT29 by Lipofectamine LTX with PLUS reagent (Invitrogen). After CBF treatment in transfected cells, the expression level of GFP was measured by POLARstar Omega.

### 2.11. Xenograft Establishment and Data Collection

To establish mouse models, 21 female BALB/c nude mice (aged 8 weeks and weighing 16–18 g) were subcutaneously injected with 5 × 10^6^ HCT116 cells. They were divided equally into three groups: intratumoural (i.t.) injection, intraperitoneal (i.p.) injection, and control group. The treatment was initiated with average tumour size of each group of about 320 mm^3^. 0.2 mg/mL injection solution was made by dissolving 10 mg of CBF in 8% of absolute ethanol and 10% of propylene glycol solution. The daily dose given to i.t. and i.p. groups was 1.5 mg/kg. When the tumour grew to 1000 mm^3^, the mouse was sacrificed. Tumour tissue specimens were collected for subsequent RNA, protein, and immunohistochemistry analysis. All experiments involving animals were approved by Griffith University (AEC No. MSC/01/08). 

### 2.12. Statistical Analysis

All results are presented as means ± S.E. A student's *t* test computation was used to analyse the data with unequal variance between each group. A *P* < 0.05 was considered significant.

## 3. Results

### 3.1. CBF Exhibits Differential Cytotoxicity in a Plethora of Cancer Cells with Potent Proapoptotic Effects on Colon Cancer

**Figure 1:**
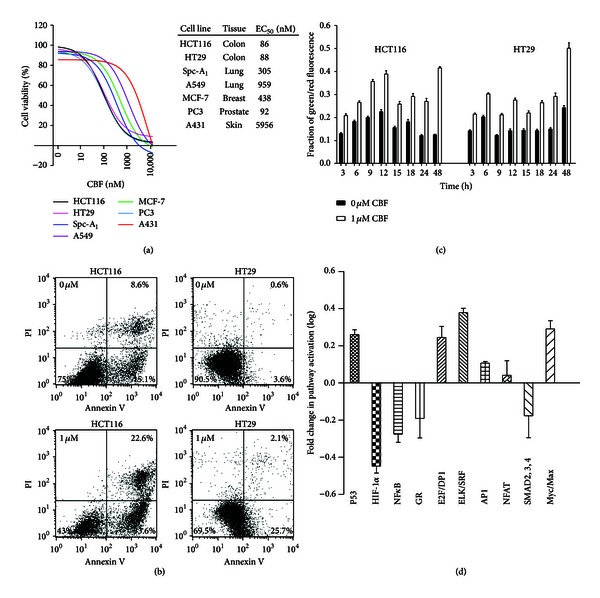
Cytotoxic effects of CBF in human cancer cell lines. (a) Cytotoxicity assays of CBF. A variety of human cancer cell lines were exposed to CBF for 24 hours and exhibited a reduction in cell numbers. The EC_50_ values were measured by using MTT assays. Results are means with standard errors from 9 replicates. (b) Annexin V/PI analysis by flow cytometry. HCT116 and HT29 cells were treated with 0 or 1 *µ*M CBF for 24 hours and stained with Annexin V/PI dyes. The lower right percentage indicated the apoptotic cells stained with Annexin V and represented a significant increment after exposure to CBF, as well as the proportion of dying cells stained with both dyes (upper right). No elevation in PI stained cells (upper left) was observed and a reduction was in living cell number (lower left) after CBF treatment. (c) The occurrence of mitochondrial permeabilisation in CBF-induced cell death. Untreated and CBF-treated HCT116 and HT29 cells were stained with JC-1 dye, which shows red in living cells and green in dying cells. The fraction (green/red) between treated and untreated cells indicated the change of mitochondrial potential transition. Results are means with standard errors from 3 replicates. (d) Deregulation in cytotoxicity signaling multipathway arrays after CBF treatment. The most significant change is in HIF-1*α*-regulated pathway, in which HIF-1*α* level was 2.8 times as low as the untreated cells. On the other hand, the most upregulation occurred in the MAPK/ERK pathway, where the expression of SRF/Elk-1 increased by 2.4 times. The logarithm of untreated cell luciferase intensity was set to baseline. p53, pathway of p53/DNA damage; HIF-1, pathway of hypoxia; NF*κ*B, pathway of NF*κ*B; GR, pathway of glucocorticoid receptor; E2F, pathway of cell cycle/pRb-E2F; SRF/Elk-1, pathway of MAPK/ERK; AP-1, pathway of MAPK/JNK; NFAT, pathway of PKC/Ca^2+^; SMAD2/SMAD3/SMAD4, pathway of TGFβ; and Myc/Max, pathway of Myc/Max. Results are means with standard errors from 4 replicates.

When colon, lung, skin, prostate, and breast cancer cell lines were treated with CBF (Figure 1(a)), significant decreases of cell viability were showed in all the cancer cell lines in a dose-dependent manner. The EC_50_ values varied from cell line to cell line indicating CBF's differential cytotoxicity. When compared with other cancer cell lines, colon cancer cell lines HCT116 and HT29 were the most sensitive to CBF. Annexin V/PI staining assays in CBF-treated HCT116 and HT29 cell lines showed that both proportions of apoptotic cells (lower right) and dying cells stained with both dyes (upper right) were significantly increased after CBF exposure (Figure 1(b)), only a very small percentage of dead cells stained positive with PI (upper left). Annexin V binds to the surface of apoptotic cells, while cell membrane-impermeant PI binds to DNA in dead cells. Our result showed a shift of cell population prone to the left, suggesting that most treated cells had undergone apoptosis rather than necrosis.

To further verify that the cells died via apoptosis, we measured the change of mitochondrial transmembrane potential using a JC-1 dye. The JC-1 dye is aggregated in mitochondrial matrix and gives out red fluorescence in healthy cells but turns to green when mitochondrial electrochemical potential alters. Therefore, a strong green signal indicates that more cells are undergoing apoptosis. As shown in Figure 1(c), mitochondrial transmembrane potential changed in both cell lines upon CBF treatment. The fractions of green over red fluorescence were elevated because of an increasing number of cells undergoing apoptosis after the addition of CBF. In addition, a study on signalling pathways underlying CBF cytotoxicity was conducted and ten cellular pathways ranging from stress, cell cycle to cell death were included. Due to low transfection efficiency of HT29, only HCT116 cells were examined by transfecting with specific luciferase reporter genes of the 10 pathways (Figure 1(d)). The differences of luciferase expression between treated and control groups indicated the activation level of the respective signalling pathway. The activity of HIF-1*α* was found to be downregulated by 2.8-fold between CBF-treated and untreated cells, while serum response factor (SRF/Elk-1) was upregulated by 2.4-fold. As such, our laboratory hypothesised that CBF plays an inhibitory role in HIF-1*α* expression but simultaneously stimulates SRF/Elk-1 mediated pathway as well in HCT116 cells.

### 3.2. Apoptotic Cell Death in HCT116 Is through Both Caspase-3-Dependent and -Independent Pathways

**Figure 2:**
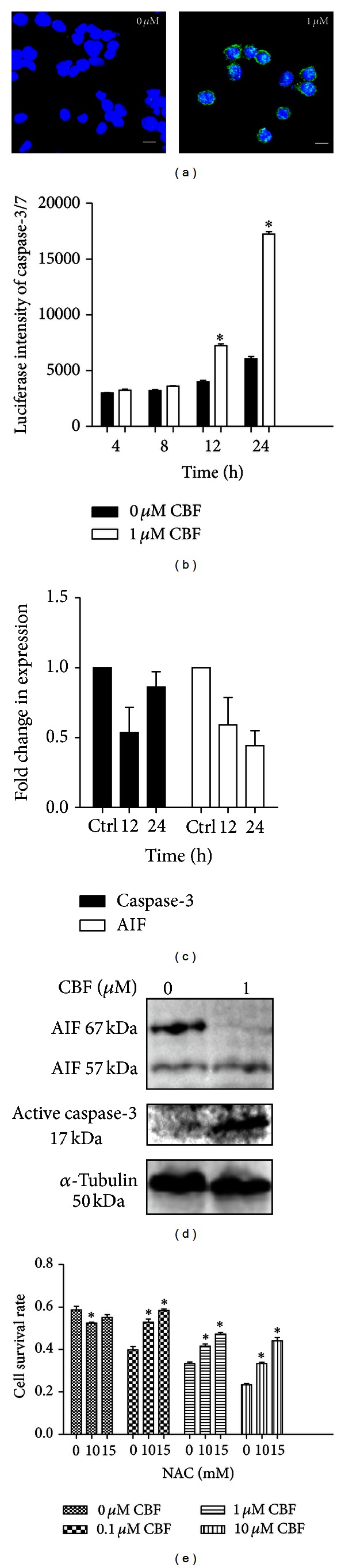
CBF-induced apoptosis is both caspase-3 dependent and independent in HCT116 cells. (a) Expression of active caspase-3 detected by confocal microscopy. HCT116 cells were treated with 0 or 1 *µ*M of CBF for 24 hours and analysed by immunofluorescence. The expression of active caspase-3 (green fluorescence) was only observed in treated HCT116. Cell nuclei were stained with DAPI (blue fluorescence). Scale bars equal 10 *μ*m. (b) Detection of caspase-3/7 activity via a time course. A significant increase of caspase-3/7 activity was found after 24-hour-CBF exposure (*N* = 9, *P < 0.05 compared to the control group). (c) RT-PCR of caspase-3 and AIF. While caspase-3 transcript levels decreased at initial 12 hours but increased at 24 hours, the mRNA level of AIF decreased by time. Results are means with standard errors from 3 replicates. (d) Protein expression of active caspase-3 and cleaved AIF after CBF exposure. After 24-hour treatment, both effective caspase-3 (17 kDa) and dissociated AIF (57 kDa) were produced. (e) Enhancement of cancer cell survival by NAC. The mixture of different concentrations of CBF and NAC partially inhibited cell death (*N* = 9, *P < 0.05).

To further explore the mode of cell death, we studied the activation of a prominent marker for apoptosis, caspase-3, by using confocal microscopy (Figure 2(a)). After 24 hours exposure to CBF, both treated and untreated cells were stained with antiactive caspase-3 antibodies. The bright green fluorescence of active caspase-3 was mostly detected in CBF-treated HCT116. Also, the temporal profile of activation caspase-3 was examined. A time course study showed that caspase-3/7 levels increased after 12 hours treatment (Figure 2(b)). Small subunit caspase-7 and large subunit caspase-3 forming a heterodimer contribute to the execution of apoptosis [25]. The intensity of active caspase-3/7 in treated cells was about three times as much as that of untreated cells after 24 hours. Interestingly, the total mRNA level of caspase-3 firstly decreased at 12 hours and then increased after 24 hours to the level of control groups (Figure 2(c)). This indicated that the cytotoxicity of CBF does not directly affect the transcription level of caspase-3. However, the level of active caspase-3 protein was upregulated (Figure 2(d)). Since mitochondrial protein AIF can lead to caspase-3-independent apoptosis, the role of AIF was further investigated in CBF-induced apoptosis. Our results showed that the mRNA level of AIF kept decreasing after CBF exposure (Figure 2(c)). This indicated that the transcription of AIF was inhibited, thereby resulting in the low expression level of total AIF (Figure 2(d)). The amount of mitochondrial-anchored AIF (67 kDa) was also diminished significantly, leaving the cleaved free AIF (57 kDa). Moreover, the addition of antioxidant NAC efficiently enhanced the resistance of HCT116 to CBF (Figure 2(e)). NAC inhibits the production of reactive oxygen species (ROS) and decreases mitochondrial potential changes, leading to reduction of AIF release. The cell viability assays showed that NAC partially elevated the survival of CBF-treated HCT116. This is most likely due to NAC preventing CBF-treated cells from mitochondrial permeabilisation and subsequent AIF-mediated caspase-3-independent apoptosis. 

### 3.3. Apoptotic Cell Death in HT29 Is through Caspase-3-Independent Pathway Only

**Figure 3:**
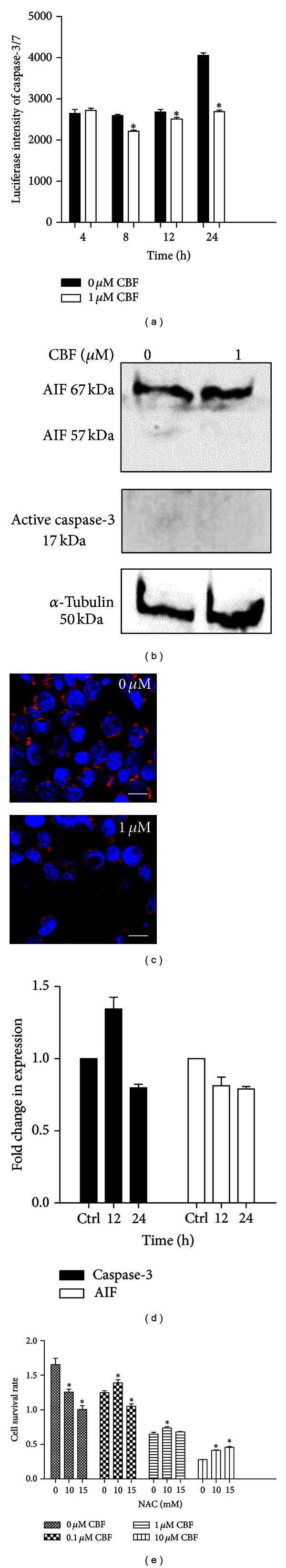
CBF-induced HT29 apoptosis is neither caspase-3 nor AIF dependent. (a) The inhibition of caspase-3/7 activity in CBF-treated HT29 cells. Treated and untreated cells were lysed and the assessment of caspase-3 luciferase intensity showed that caspase-3/7 activity was significantly downregulated after 24-hour treatment (*N* = 9, *P < 0.05). (b) No expression of active caspase-3 and cleaved AIF. After 48-hour treatment, only mitochondria-localised AIF (67 kDa) was detected by immunoblotting. (c) No obvious shift in AIF distribution between treated and control groups. After CBF addition of 24 hours, red fluorescence representing AIF expression exhibited no enhancement of AIF nuclear translocation. Cell nuclei were stained with DAPI (blue fluorescence). Scale bars equal 10 *μ*m. (d) RT-PCR of AIF and caspase-3. mRNA level of caspase-3 firstly increased after 12 hours and then decreased at 24 hours, but there was no significant change in AIF mRNA level. Results are means with standard errors from 3 replicates. (e) Failure of cancer cell rescue by NAC. The mixture of CBF and NAC was unable to prevent cells from CBF-induced apoptosis (*N* = 9, *P < 0.05).

In contrast to HCT116, the activity of caspase-3/7 was reduced in HT29 cells after 24 hours of CBF exposure (Figure 3(a)). No active caspase-3 or cytosolic AIF (57 kDa) was detected even after 48 hours of CBF treatment (Figure 3(b)). Consistently, no significant shift of AIF intracellular distribution between 48-hour-treated and untreated HT29 cells was observed by confocal microscopy (Figure 3(c)). Analysis of mRNA level showed no apparent involvement of CBF in transcription of caspase-3 and AIF (Figure 3(d)). The mRNA level of caspase-3 increased at 12 hours but eventually decreased after 24 hours, while AIF mRNA level was slightly reduced after CBF addition. Furthermore, in contrast to the case of HCT116, NAC was unable to counter the CBF cytotoxicity of HT29 cells until the CBF concentration of up to 10 mM (Figure 3(e)), indicating that ROS accumulation in mitochondria is not a direct cause of the cell death. In addition, the high sensitivity of untreated HT29 to NAC alone was most likely to result from suppression of c-Src phosphorylation [26]. The massive blockade of c-Src kinases by NAC arrests HT29 cells in G1 phase of cell cycle. 

### 3.4. The Inhibition of HIF-1*α* Protein by CBF Is Not due to the Suppression of RNA Transcription

**Figure 4:**
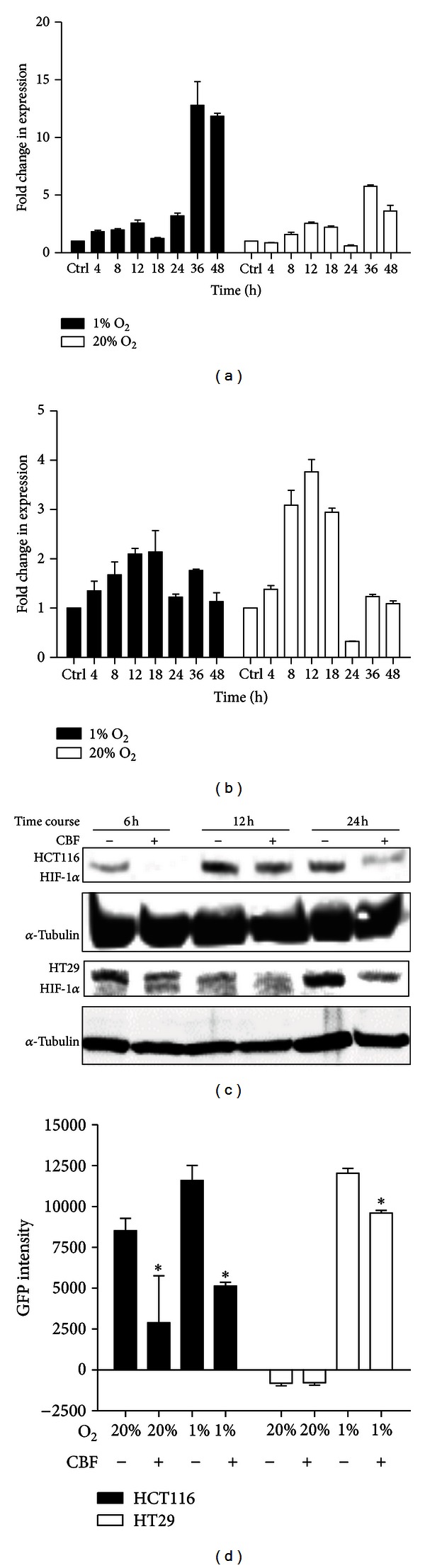
CBF affects HIF-1*α* transcription and protein expression. (a) Elevation of HIF-1*α* transcription level by CBF in HCT116 cells. Compared with that in normal atmosphere, the level of HIF-1*α* mRNA doubled when HCT116 cells were in lack of oxygen. (b) Elevation of HIF-1*α* transcription level by CBF in HT29 cells. Dramatic upregulation of HIF-1*α* mRNA was only from 8 to 18 hour time points, followed by a sharp fall at 24 hours. The patterns of HIF-1*α* transcription of HT29 between 1% and 20% were similar. (c) Inhibition of HIF-1*α* protein expression. Under hypoxic (100 *µ*M CoCl_2_) conditions, HIF-1*α* protein expression was suppressed by CBF at different time points. (d) Inhibition of GFP fused HIF-1*α* expression. Plasmid pGFP-HIF-1*α* was transfected into HCT116 and HT29 cells, followed by a 24-hour-CBF treatment under 1% or 20% oxygen. Compared with the control groups, 1 *μ*M of CBF exposure effectively inhibited the expression of GFP under hypoxic conditions (*N* = 6, *P < 0.05).

Unlike the result from multipathway reporter arrays, HIF-1*α* mRNA levels of CBF-treated HCT116 and HT29 cells noticeably increased during 48 hours under hypoxic and normoxic conditions, compared with that of controls (Figures 4(a) and 4(b)). This result suggested that transcription of HIF-1*α* mRNA is relevant to CBF exposure, and CBF particularly enhances HIF-1*α* transcription under 1% oxygen atmosphere. However, further investigation on HIF-1*α* protein showed that the expression of HIF-1*α* protein was inhibited by CBF (Figure 4(c)). The most significant inhibition was at 24 hours time point in both cancer cell lines. Another method to assess the level of HIF-1*α* protein was by measuring the expression of a GFP-HIF-1*α* fusion protein in CBF-treated HCT116 and HT29 cells. Transfected cells were exposed to CBF for 24 hours under 1% of oxygen. GFP intensity under 20% of oxygen was also measured and predictably at low levels. As shown in Figure 4(d), the expression level of GFP in CBF-treated HCT116 and HT29 cells was significantly reduced.

### 3.5. CBF Affects the Expression of HIF-1*α* in HCT116 Tumour Bearing Xenografts

**Figure 5:**
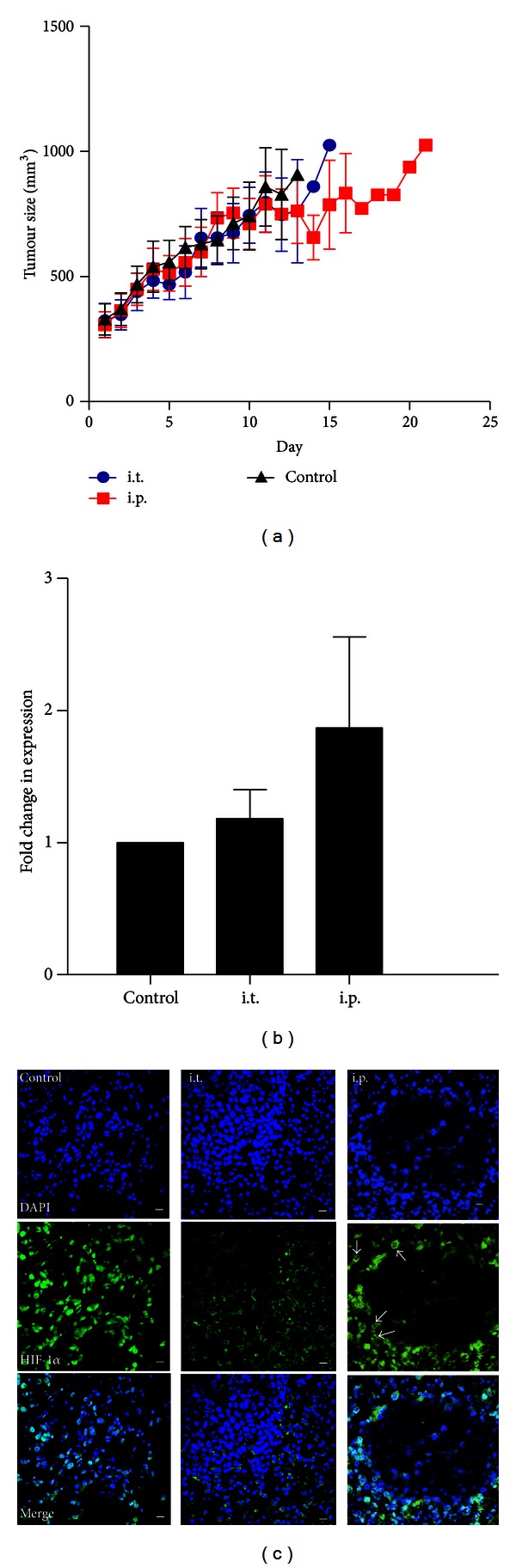
Analysis of CBF treatment in HCT116 implanted mouse models and tumour tissue samples. (a) Tumour size versus CBF treatment. The lowest tumour growth rate was found in i.p. group during CBF treatment. (b) Analysis of HIF-1*α* mRNA level in tumour tissues. There was a significant upregulated HIF-1*α* level in i.p. group. Results are means with standard errors from 4 replicates. (c) Inhibition of HIF-1*α* nuclear translocation in the i.t. sample. CBF strongly inhibited HIF-1*α* nuclear accumulation in the tumour of xenografts implanted with HCT116 cells. Scale bars equal 10 *μ*m. White arrows: HIF-1*α* in the cytosol.

In an effort to define the anticancer function of CBF in animal models, we established HCT116 implanted xenografts, and CBF treatment (1.5 mg/kg) was given daily by i.t. and i.p. injection. The lowest tumour growth rate was in i.p. group (Figure 5(a)). All mice from groups of control and i.t. injection were sacrificed on day 13 and day 15, respectively. The mRNA analysis of tumour tissues showed that HIF-1*α* mRNA level was dramatically elevated in i.p. group (Figure 5(b)). This finding is consistent with the increase of HIF-1*α* mRNA level in cell line experiments. More significantly, the fluorescent images from mouse tissues exhibited a clear inhibition in nuclear translocation of HIF-1*α* in i.t. group (Figure 5(c)). This shift was also found in a number of cells in i.p. group. Compared with treatment groups, the HIF-1*α* import was apparent in the control group.

## 4. Discussion

Natural medicine, like Chansu, has been widely used in the clinic for human patients in China, Japan, and other Asian countries as an anaesthetic, antibiotic, anticancer and cardiotonic medication. However, like many of these natural therapies, their molecular mechanisms have not been elucidated. As such the claims of effectiveness have not been fully tested. Recently, an increasing number of *in vitro* studies have revealed the proapoptotic function of Chansu and its components in cancer cells [14, 16, 17]. CBF is one of the major bufadienolide compounds and a variety of molecular mechanisms have been proposed, that is, inhibition of nuclear factor Kappa B (NF*κ*B) activation [18] and attenuation of phosphorylation in ERK-related pathway [27]. According to the classification of bufadienolides, CBF is also a type of CGs. One distinct advantage of CG compounds as anticancer agents is that they have high efficacy and safety profiles [24, 28, 29]. This is consistent with the finding of our laboratory that the human head and neck cancer cell line HN5 was drastically resistant to CBF-induce cell death (EC_50_ > 6.5 *µ*M, Li and Wei, publication in preparation). We showed that CBF inhibitory effects on cancer cell growth were variable, with different EC_50_ values among different cell lines. The lowest EC_50_ exhibited at 86 nM in HCT116 and the most resistance was in A431 at approximately 6 *μ*M of CBF. Cell viability assays showed that colon cancer cell lines exhibited an overall higher cell death efficacy of CBF than that of breast, skin, and lung cancer cell lines. This high sensitivity to colon cancer cells is consistent with the previous finding of Sakai et al. [30], who demonstrated that ouabain has a preferential binding to alpha 3 (*α*3) subunit of Na^+^/K^+^-ATPase. They showed that there was an elevation of *α*3 subunit expression in colorectal cancer tissues, as well as in the HT29 cell line. Therefore, it is possible that CBF selectively binds to *α*3 subunits and gives rise to a high inhibitory effect in colon cancer cell lines. Furthermore, we found that even within two highly sensitive cancer cell lines, HCT116 and HT29 cells underwent very distinct apoptotic processes. The classic caspase-3-dependent apoptosis was only detected in CBF-treated HCT116 but not in CBF-treated HT29.

Recent reports suggest that another key feature of the CG-related proapoptotic mechanism is mitochondrial injury [31]. Indeed the results of this study demonstrated that the change of mitochondrial potential is involved in CBF-induced cell death and linked this change to AIF-mediated apoptosis. The release of AIF can be due to a sustained increase of intracellular Ca^2+^ level, which causes calpain activation [32]. Calpain is a calcium-dependent protease and activated calpain cleaves AIF in mitochondria [33]. Yeh et al. revealed an elevation of cytosolic Ca^2+^ in prostate cancer cell lines after CBF treatment [20]. This mechanism could also operate in AIF-regulated apoptosis in HCT116 cells. Apart from calpain cleavage, a high intracellular Ca^2+^ concentration also leads to the production of ROS in mitochondria [34]. Such enhancement of ROS by CBF is a likely explanation for the disruption of mitochondria in our experiments. Although the expression of dissociated AIF was only observed in CBF-treated HCT116 cells, increased mitochondrial permeabilisation was observed in both cell lines. Moreover, Newman et al. (2006) found that NAC prevents oleandrin (a type of CG) induced mitochondrial condensation in malignant melanoma by inhibition of ROS production [31]. The addition of NAC to HCT116 cells also significantly reduced CBF-induced apoptosis in our experiments. This further indicated a likely correlation between mitochondrial disruption and CBF cytotoxicity. Intriguingly, proapoptotic Bax expression was suppressed after CBF treatment in both colon cancer cells (see Supplementary Figure  1 available online at http://dx.doi.org/10.1155/2013/849054). After all, CBF did elicit apoptosis in HT29, but the apoptotic pathways remain independent of the activity of caspase-3 and AIF, suggesting other apoptotic mechanisms could be involved in CBF anti-cancer capacity. A possibility could be through necroptosis, which is a necrotic mechanism crosslinking to apoptosis [35]. Necroptosis depends on the activation of receptor-interacting protein kinases 1 (RIPK1) and RIPK3, which leads to mitochondrial disrupt due to several factors, such as ATP depletion, ROS accumulation and elevation of Ca^2+^ [36]. Another important characteristic of necroptosis is suppression of caspase-8, a precursor of caspase-3/7 [37]. Thus, necroptosis could be a rational explanation for mitochondrial injury and caspase-3 deactivation in CBF-induced cell death in HT29 cells. In addition, AIF possesses a capacity to regulate caspase-independent necroptosis, but it is not essential [38]. Our results implied no involvement of AIF activity in CBF-treated HT29 cells.

Furthermore, the assessment of the 10-pathway reporter arrays suggested a multifactorial network involved in CBF-mediated apoptosis. Overall, the most significant inhibition was in HIF-1*α*-regulated pathway. The expression of HIF-1*α* intimately corresponds to changes in oxygen levels [39]. During oxygen deprivation, degradation of HIF-1*α* is inhibited, permitting HIF-1*α* to enter the nucleus, where it binds to aryl hydrocarbon receptor nuclear translocator and initiates the transcription of hypoxia-responsive genes. HIF-1*α* mediates the transcription of several oncogenic genes, especially vascular endothelial growth factor (VEGF) and thereby promotes cell proliferation [40]. In relation to the inhibition of HIF-1*α*, although there is no direct relationship established between HIF-1*α* and CBF, several previous studies showed that digoxin and other cardiac glycosides inhibit HIF-1*α* synthesis to retard tumour growth [41, 42]. Thus, there is a possibility that the digoxin-like molecule CBF reduces HIF-1*α* levels in colon cancer cell lines as well. Indeed our findings showed that HIF-1*α* protein expression was suppressed by CBF as early as 6 hours after treatment in both colon cancer cell lines, regardless of the elevation of mRNA transcript. Another result from the estimation of GFP-HIF-1*α* expression confirmed further the inhibition of exogenous HIF-1*α* expression in the presence of CBF. Moreover, HIF-1*α* was found to localise in the cytosol of mouse tumour cells and did not accumulate in the nucleus. Although there is little information in hand to elucidate the mechanism involved in CBF function in tumour tissues, an inhibitory role was still revealed in the blockade of HIF-1*α* import in CBF-treated xenografts implanted with HCT116. Apart from the protein downregulation, the increase of HIF-1*α* mRNA level might be due to the activation of MAPK/ERK pathway, the second most affected pathway shown in 10-pathway reporter assays. A number of previous studies have revealed that stimulation of the MAPK pathway enhances HIF-1*α* transcriptional activity [43]. Thus, taken together our findings suggest that the hypoxia-regulated cellular pathway is involved in CBF-driven cell death. 

## 5. Conclusion

CBF inhibited cancer cell growth by induction of apoptosis through different pathways. The significant inhibition of HIF-1*α* nuclear translocation strongly suggested the involvement of hypoxia pathway *in vivo *in animal models. Taken together, our findings showed that CBF is a highly potent inhibitor of cancer growth, has high efficacy and safety profiles, and has the potential to be developed as an effective anticancer chemotherapeutic agent.

## Supplementary Material

The expression of proapoptotic protein Bax was significantly inhibited in treated HCT116 and HT29 cells.
