# Supplementary material for: The Mechanisms of Chansu in Inducing Efficient Apoptosis in Colon Cancer Cells
Source: Evid Based Complement Alternat Med. 2013 May 30;2013:849054. doi: 10.1155/2013/849054 (PMC3683424; doi:10.1155/2013/849054)
Supplement: Supplementary file 1 — The expression of proapoptotic protein Bax was significantly inhibited in treated HCT116 and HT29 cells. [file 849054.f1.pptx]

## Slide 1
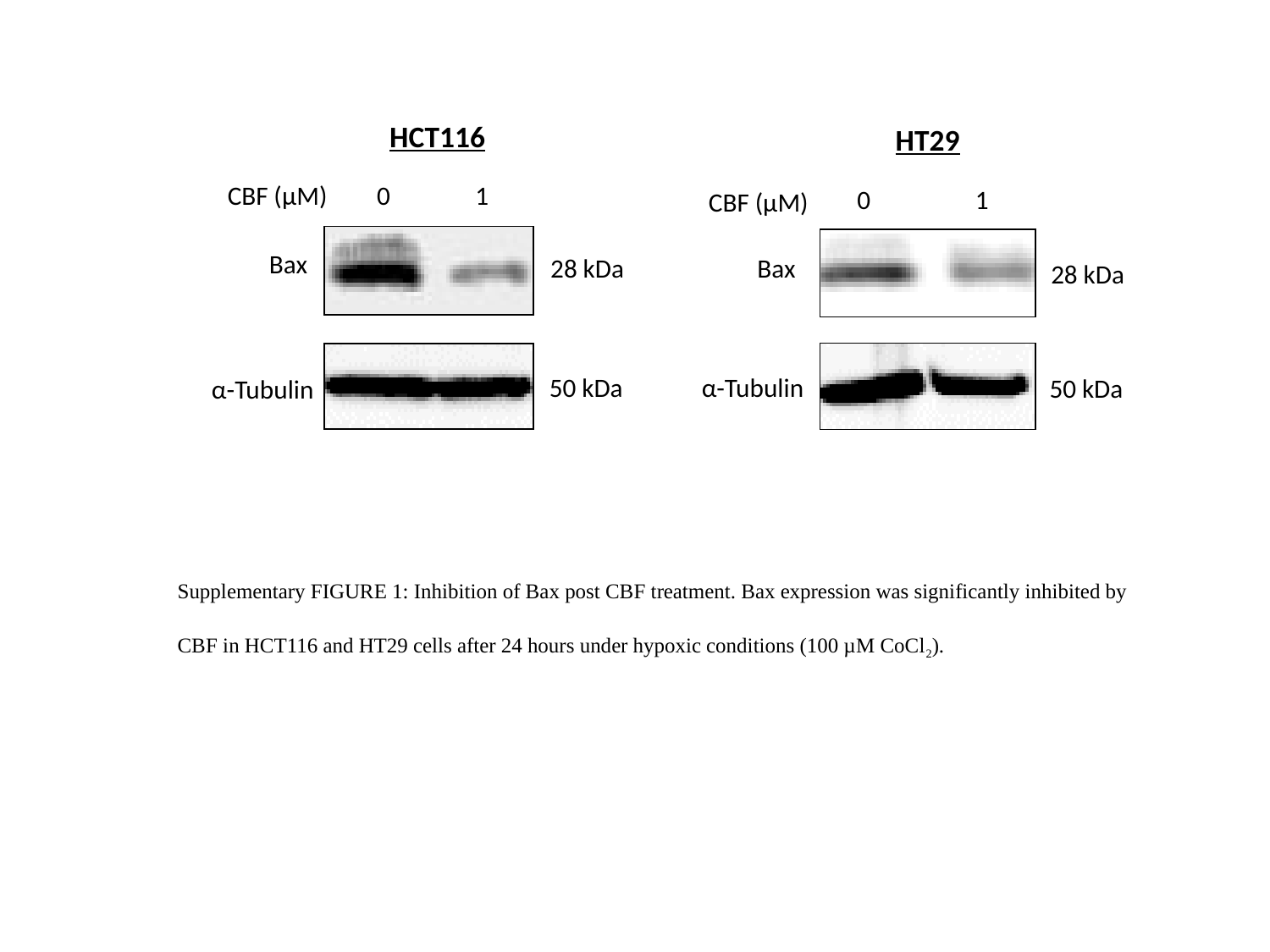

HCT116
HT29
CBF (μM)
0
1
0
1
CBF (μM)
Bax
28 kDa
Bax
28 kDa
α-Tubulin
50 kDa
50 kDa
α-Tubulin
Supplementary FIGURE 1: Inhibition of Bax post CBF treatment. Bax expression was significantly inhibited by CBF in HCT116 and HT29 cells after 24 hours under hypoxic conditions (100 µM CoCl2).
